# Cancer comorbidity in patients with non-obstructive coronary artery disease: Depressive symptoms related to C-reactive protein

**DOI:** 10.1016/j.bbih.2020.100088

**Published:** 2020-05-30

**Authors:** Dounya Schoormans, Jos W. Widdershoven, Paula M.C. Mommersteeg

**Affiliations:** a CoRPS - Center of Research on Psychological and Somatic Disorders, Department of Medical and Clinical Psychology, Tilburg University, Tilburg, the Netherlands; b Department of Cardiology, Elisabeth-Tweesteden Hospital, Tilburg, the Netherlands

**Keywords:** Depression, Inflammation, hsCRP, Non-obstructive artery disease, Cancer survivors, Cardio-oncology

## Abstract

**Background:**

Based on guidelines for cardiovascular risk assessment among non-cancer populations, depression and anxiety can be seen as risk factors for CVD, on top of cardiotoxic cancer treatment and traditional CVD risk factors among cancer survivors. Increased inflammation can be a shared potential pathophysiological mechanism, as higher levels of inflammation (like C-reactive protein, CRP) are known associates of depression and anxiety. In turn, increased inflammation is involved in the pathogenesis of CVDs. Furthermore, both cancer and cancer treatment including chemotherapy and radiation can lead to elevated levels of inflammation. We will therefore examine whether the relation between depression and anxiety with inflammatory markers among patients with either CVD or cancer is different from those with both conditions.

**Method:**

The TweeSteden Mild Stenosis (TWIST) study among patients with non-obstructive coronary artery disease (NOCAD, luminal narrowing <60%), a type of ischemic heart disease, previously reported a significant association between depressive symptoms and increased inflammation (measured by high-sensitive (hs)CRP). Of the included NOCAD-patients, 6% had a history of cancer. The TWIST patient sample was therefore used to explore whether the association between depression and elevated inflammation (hsCRP) was similar for NOCAD-patients with and without a history of cancer.

**Results:**

The association between depressive symptoms and increased hsCRP levels is stronger among NOCAD-patients with a history of cancer than among NOCAD-patients without a history of cancer. Furthermore, whereas this relation is mediated by lifestyle factors among NOCAD-patients without cancer, the association remained significant after adjusting for BMI, smoking, and physical activity among NOCAD-patients with a history of cancer.

**Conclusion:**

The stronger association between depression and hsCRP among NOCAD-patients with a history of cancer indicates that there may be an additive or synergistic effect of having NOCAD and cancer for general inflammation and possibly depression.

## Highlights

• Depression and anxiety as potential risk factors for CVD among cancer patients.
• Depressive symptoms are related to increased hsCRP levels among patients ischemic disease.
• Depressive symptoms are stronger related to increased hsCRP among patients with ischemic disease and cancer.
• Among ischemic patients with cancer, the association is not mediated by lifestyle factors.

## 1 Introduction

Cardiovascular disease (CVD) is the number one co-morbidity in cancer patients and responsible for half of all non-cancer related mortalities (Aziz, 2007). A multiple-hit hypothesis has been proposed in cardio-oncological research explaining increased CVD risk among cancer survivors (Jones et al., 2007). It describes that survivors are exposed to a series of sequential or concurrent events that together make them vulnerable to develop CVD. According to the multiple-hit hypothesis CVD risk increases with cancer diagnosis, and receiving cardiotoxic cancer treatment (first hits) and traditional CVD risk factors (subsequent hit). (Jones et al., 2007).

**Fig. 1:**
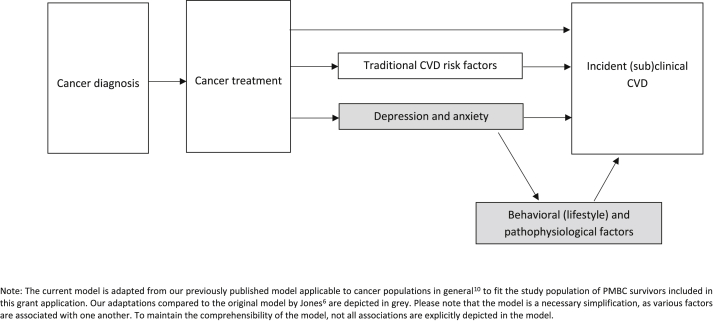
The adapted multiple-hit hypothesis: the psychological factors depression and anxiety as an additional ‘hit’ in the pathogenesis of CVD and underlying behavioural and pathophysiological mechanisms. Note: Our adaptations compared to the original model by Jones et al. are depicted in grey. Please note that the model is a necessary simplification, as various factors are associated with one another. To maintain comprehensibility of the model, not all associations are explicitly depicted in the model.

Current clinical guidelines for cardiovascular risk assessment among non-cancer populations highlight the importance of depression and anxiety as risk factors, with estimates at the same level as traditional CVD risk factors such as hypertension, due to consistent findings that these psychological factors increase the risk for incident CVD (Lichtman et al., 2014). Research from the psycho-oncology field has shown that up to 50% of cancer survivors experience symptoms of depression and anxiety as a consequence of cancer diagnosis and treatment (Linden et al., 2012). Combining knowledge of psycho-cardiology and psycho-oncology led to our adaptation of the multiple hit hypothesis, see Fig. 1 (Schoormans et al., 2016). The potential impact of depression and anxiety as risk factors for CVD was added to the existing hits, introducing the novel field of psycho-cardio-oncology. We suggested increased inflammation as shared potential pathophysiological mechanism, as higher levels of inflammation (like C-reactive protein, CRP) are known associates of depression and anxiety (Valkanova et al., 2013). In turn, increased inflammation is involved in the pathogenesis of CVDs, such as atherosclerosis. Furthermore, both cancer and cancer treatment including chemotherapy and radiation can lead to elevated levels of inflammation (Schaue et al., 2015; Tsavaris et al., 2002). It can therefore be hypothesized that the relation between depression and anxiety with inflammatory markers among patients with either CVD or cancer may be different from those with both conditions. However, to date no studies have been reported examining the relation between depression and anxiety with inflammatory markers among patients with both cancer and CVD.

The TweeSteden Mild Stenosis (TWIST) study among patients with non-obstructive coronary artery disease (NOCAD, luminal narrowing <60%), a type of ischemic heart disease, previously reported a significant association between depressive symptoms and increased inflammation (measured by high-sensitive (hs)CRP) (Mommersteeg et al., 2014). The authors concluded that the found association was (partially) mediated by lifestyle factors (i.e. smoking, BMI, and physical activity). Of the included NOCAD-patients, 6% had a history of cancer. The TWIST patient sample therefore allows us to explore whether the association between depression and elevated inflammation (hsCRP) was similar for NOCAD-patients with and without a history of cancer. We hypothesise that NOCAD-patients who have a history of cancer show a stronger association between depression and inflammation (hsCRP) than NOCAD-patients without cancer.

## 2 Materials and method

Details on data collection have been published elsewhere (Mommersteeg et al., 2014). In short, NOCAD-patients were included if visible wall irregularities were present in the absence of obstructive (<60%) stenosis of a coronary artery, but less than 50% obstruction of the left main coronary artery based on coronary angiography (CAG) or multislice computed tomography (CT)-scan (Mommersteeg et al., 2014). Questionnaires were sent and returned by postal mail, information was collected from patient hospital records, and patients had their blood samples taken at the regular blood collection services of the hospital. Ethical approval was obtained from the local Medical Ethics Committee (NL22258.008.08). The study was performed in accordance with the declaration of Helsinki, and registered as an observational cohort study at ClinicalTrials.gov (NCT01788241).

### 2.1 Measurements

Depressive symptoms (named ‘depression’; HADS-D) and anxiety (HADS-A) were measured with the two 7-item subscales of the Hospital Anxiety and Depression Questionnaire, with scores ranging from 0 to 21. Blood was collected in 10ml vacutainers for routine clinical assessment of hsCRP in serum using the Cobas c502 analyzer. CAD-disease severity was categorized as number of vessels with wall irregularities indicative of stenosis. Diagnostic source was dichotomized into patients being included via CAG or CT. Information on comorbid conditions were extracted from medical records. Those with a previous cancer diagnosis were categorized as NOCAD-patient with a history of cancer versus those without. Additionally the number of co-morbidities (not including cancer) were coded as none, one or two, or more co-morbidities. Cardiac medication was categorized and recoded into a medication count variable (0–7). Self-report and medical records were used to determine BMI and current smoking status. Physical activity was measured with one item and recoded into being ‘active’ or ‘inactive’.

### 2.2 Statistical analyses

HsCRP data were ln-transformed. Patient characteristics stratified by NOCAD-patients with versus without cancer were compared using chi-square or t-tests. To examine whether the association between depression or anxiety with hsCRP was similar for NOCAD-patients with and without a history of cancer hierarchical multivariable regression analyses were run with the interaction term cancer∗depression/anxiety and its main effects. Covariates were added to the model in four additive blocks (Mommersteeg et al., 2014). First, adjusting for age en gender (M1). Second, the sociodemographic factors: living with a partner, and education (college/higher vs. high school/lower) were added (M2). Third, clinical characteristics were added; disease severity, diagnostic source, cardiac medication, and number of co-morbidities (M3). Finally, the lifestyle variables; BMI, smoking status, and physical activity were added (M4). Two-sided p-value of 5% was considered statistically significant using SPSS 24.0.

## 3 Results

In total, 547 NOCAD-patients were included (mean age 61 years, 52% women). Thirty-three patients (6%) had a history of cancer. NOCAD-patients with compared to those without a history of cancer were on average older (66 years vs 61 years). With respect to the other covariates (gender, sociodemographics, and lifestyle factors) groups did not differ. Moreover, scores on hsCRP, depression and anxiety were not different between both groups.

Multivariable analyses showed a significant interaction for depression with cancer in all four models (Table 1). Relating anxiety to hsCRP showed no significant main or interaction effects (cancer∗anxiety), Table 1.

**Table 1:** Multivariable hierarchical regression analyses independently relating depression and anxiety to hsCRP.

	hsCRP^§^	
	Depression	Anxiety
Model 1: Age, sex		
Depression/Anxiety	Beta ​= ​0.14, p ​< ​0.01∗∗	Beta ​= ​0.08, p ​= ​0.14
Depression/Anxiety∗cancer	Beta ​= ​0.10, p ​= ​0.05	Beta ​= ​0.01, p ​= ​0.85
F change, adj R2	FΔ ​= ​4.26, adjR^2^ ​= ​0.04, p ​< ​0.01∗∗	FΔ ​= ​1.86, adjR^2^ ​= ​0.01, p ​= ​0.101
**Model 2: Sociodemographics**		
Depression/Anxiety	Beta ​= ​0.10, p ​< ​0.05∗	Beta ​= ​0.04, p ​= ​0.45
Depression/Anxiety∗cancer	Beta ​= ​0.10, p ​= ​0.04∗	Beta ​= ​0.02, p ​= ​0.72
F change, adj R2	FΔ ​= ​3.04, adjR^2^ ​= ​0.05 p ​< ​0.01∗∗	FΔ ​= ​4.28, adjR^2^ ​= ​0.03, p ​= ​0.01∗∗
**Model 3: Clinical characteristics**		
Depression/Anxiety	Beta ​= ​0.07, p ​= ​0.16	Beta ​= ​0.01, p ​= ​0.89
Depression/Anxiety∗cancer	Beta ​= ​0.12, p ​= ​0.02∗	Beta ​= ​0.04, p ​= ​0.46
F change, adj R2	FΔ ​= ​2.90, adjR^2^ ​= ​0.07, p ​< ​0.01∗∗	FΔ ​= ​3.03, adjR^2^ ​= ​0.05, p ​< ​0.01∗∗
**Model 4: Lifestyle**		
Depression/Anxiety	Beta ​= ​0.04, p ​= ​0.50	Beta ​= ​−0.01, p ​= ​0.91
Depression/Anxiety∗cancer	Beta ​= ​0.11, p ​= ​0.02∗	Beta ​= ​0.04, p ​= ​0.48
F change, adj R2	FΔ ​= ​7.78, adjR^2^ ​= ​0.11, p ​< ​0.01∗∗	FΔ ​= ​8.63, adjR^2^ ​= ​0.10, p ​< ​0.01∗∗

Note: Model 1: adjusted for the covariates age and sex; Model 2, adjusted for the covariates of model 1 + sociodemographics: living with a partner, and educational level; Model 3, adjusted for the covariates of model 2 + clinical characteristics: disease severity, diagnostic source, cardiac medication, and number of co-morbidities; Model 4, adjusted for the covariates of model 3 + lifestyle factors: BMI, current smoking status, and physical activity. ∗p ​< ​0.05, ∗∗p ​< ​0.01; FΔ ​= ​F change; adjR^2^ ​= ​adjusted R squared. ^§^ ​= ​ln transformed hsCRP.

Exploratory stratified analyses were performed repeating multivariable hierarchical analyses for those with and without a history of cancer separately (Table 1). Given the limited number of NOCAD-patients with cancer, covariates of the four blocks were examined in sequence. Among NOCAD-patients with a history of cancer, depression was related to higher hsCRP after controlling for covariates in each of the separate blocks. That is, after adjustment for age and sex (beta ​= ​0.66, p ​< ​0.01); for sociodemographics (beta ​= ​0.53, p ​< ​0.01); for clinical characteristics (beta ​= ​0.54, p ​< ​0.01) or for lifestyle factors (beta ​= ​0.70, p ​= ​0.05). Results showed that among those without a history of cancer; depression was related to higher hsCRP levels after adjustment for age and sex (beta ​= ​0.13, p ​= ​0.01). However, the association was not significant after adjustment for sociodemographics (beta ​= ​0.09, p ​= ​0.10), for clinical characteristics (beta ​= ​0.07, p ​= ​0.23) or for lifestyle factors (beta ​= ​0.06, p ​= ​0.21).

## 4 Conclusion

The association between depressive symptoms and increased hsCRP levels is stronger among NOCAD-patients with a history of cancer than among NOCAD-patients without a history of cancer. Furthermore, whereas this relation is mediated by lifestyle factors among NOCAD-patients without cancer, the association remained significant after adjusting for BMI, smoking, and physical activity among NOCAD-patients with a history of cancer. The stronger association between depression and hsCRP among NOCAD-patients with a history of cancer indicates that there may be an additive or synergistic effect of having NOCAD *and* cancer for general inflammation and possibly depression. This could be due to cancer (treatment) increasing inflammatory levels (Schaue et al., 2015; Tsavaris et al., 2002) or elevated levels of depression among this subsample. However, in our sample no main group differences were observed. A previous study has reported that associations between depression and inflammation were present only among those with a history of childhood trauma (Han et al., 2016). It can be hypothesized that experiencing major trauma like getting cancer make patients more vulnerable to alterations to their immune system.

Results of this study should be interpreted in light of the following limitations. First, only 33 patients had a history of cancer. Nevertheless, different associations for both subsamples were reported. Furthermore, no clinical cancer characteristics were available like type of cancer diagnosis, years since cancer diagnosis, and received cancer treatment, whereas we know that these factors increase levels of inflammation (Schaue et al., 2015; Tsavaris et al., 2002). Third, we examined a single marker of general inflammation, hsCRP, instead of other known associates of depression, like up-stream pro-inflammatory cytokines. We did control for several known relevant covariates and found an association between depression and hsCRP that was not mediated by sociodemographic, clinical characteristics, or lifestyle factors among NOCAD-patients with a history of cancer. This finding supports the suggested inflammatory mechanism underlying the relation between depression and CVD risk among cancer survivors as previously presented in the adapted multiple-hit hypothesis (see Fig. 1) explaining CVD risk among oncological patients (Schoormans et al., 2016).

## Funding

None.

## Declaration of competing interest

The authors declare that they have no known competing financial interests or personal relationships that could have appeared to influence the work reported in this paper.
